# Postpartum psychosis in peripartum cardiomyopathy: a case report

**DOI:** 10.1186/s12888-020-02522-2

**Published:** 2020-03-11

**Authors:** Odhiambo Henry Owuor, James Amisi Akiruga, Jeremiah Laktabai, Samuel Ateya, Salwa Mohamed Omar

**Affiliations:** School of Medicine, Department of Family Medicine, Moi University, P. O. Box 3900-30100, Eldoret, Kenya

**Keywords:** Peripartum cardiomyopathy (PPCM), Postpartum psychosis, Sudden cardiac death, QTc prolonfation

## Abstract

**Background:**

This case report highlights the rare occurrence of postpartum psychosis in the setting of peripartum cardiomyopathy, which can have rare presentations like arrhythmias and pulmonary edema; and the challenges one should anticipate while managing these conditions together. Caution is advised whenever antipsychotic drugs are to be administered to a patient with a cardiac condition as these drugs potentially increase the risk of arrhythmias and sudden death.

**Case presentation:**

A 35 year old grand multiparous woman who was 1 week into puerperium was admitted with severe difficulty in breathing at rest, chest congestion and pain. She also had easy fatigability, orthopnea, paroxysmal nocturnal dyspnea, edema, tachycardia, tachypnea, irregularly irregular heart rate with a pulse deficit, elevated jugular venous pressure, cardiomegaly, hepatomegaly and pulmonary crepitations. On the sixth day while improving on standard drugs for heart failure, she developed bizarre behavior and confusion. She also had auditory, visual and olfactory hallucinations; violence to the baby and the husband; and refusal to feed and take medication. There was no altered sensorium and the vital signs were normal. She was diagnosed with puerperal psychosis during the management of peripartum cardiomyopathy.

**Conclusion:**

In the rare occurrence of puerperal psychosis in the course of management of peripartum cardiomyopathy one must be acutely aware of the risk of sudden cardiac death occasioned by use of antipsychotics, either directly or due to arrhythmias. Continuous electrocardiogram (ECG) monitoring or use of alternative management modalities is thus highly advised.

## Background

Peripartum cardiomyopathy (PPCM) is a rare and idiopathic, heart failure that develops in the last month of pregnancy or up to 5 months postpartum with left ventricular systolic dysfunction [1, 2]. It usually presents with symptoms of congestive heart failure e.g. dyspnea on exertion, orthopnea, paroxysmal nocturnal dyspnea, and edema of the lower extremities [3]. However, less common presentations include arrhythmias, pulmonary edema and thromboembolism [4–6]. PPCM has a good prognosis as up to four-fifths of women with PPCM recover to normal range left ventricular systolic function within the first 6 months [3]. A significant proportion of mortality is attributed to arrhythmias which are thought to be responsible for more than a quarter of deaths in women with PPCM [6]. Standard treatments for heart failure with reduced ejection fraction are indicated in PPCM, with special attention to avoiding adverse fetal effects in women who are still pregnant [7].

Postpartum, or puerperal psychosis (PP) is a psychiatric emergency that affects a small proportion of women shortly after childbirth [8]. It presents with hallucinations, delusions, cognitive disorganization, confusion, anxiety and sleep problems. Occasionally affected mothers may attempt to injure themselves or their child, with resultant maternal suicide or infanticide [9, 10]. The major risk factor is history (personal or familial) of bipolar disorder or related psychotic disorder [9]. Environmental factors, particularly obstetric complications, are also implicated as precipitators [9, 11]. Acute treatment involves use of a mood stabilizer (e.g., lithium, valproic acid, and carbamazepine) in combination with antipsychotic medications and benzodiazepines [10, 12]. Antipsychotic medications are however known to increase corrected QT interval (QTc) in a dose-dependent manner as a class, same as some of the medications used in the acute management of PPCM [13]. There is even the possibility of synergy with worse effects on the QT [13]. QT prolongation is associated with sudden cardiac death (SCD) [13, 14].

The aim of this case report is to highlight the rare occurrence of postpartum psychosis in the course of management PPCM and the challenges its (PPs) management poses to that of PPCM and vice versa. There is the risk posed by use of antipsychotic drugs in any patient with a cardiac condition, including the potential increase of arrhythmias and sudden death.

## Case presentation

A 35 year old African female was admitted to the emergency unit with difficulty in breathing, chest congestion and chest pain for 3 days. The para 6 + 1 woman had delivered vaginally a week prior to presentation. Her pregnancy and delivery were uneventful. She had been well prior to the development of the difficulty in breathing which was insidious beginning the fourth day after delivery. As it worsened, it was associated with difficulty in completing sentences and walking even a few steps. At the time of admission she had dyspnea at rest, which worsened on lying flat. The night before admission, she had episode of waking up gasping for breath. She reported that her legs were also swollen. The chest congestion and chest pain had also developed progressively. The pain mostly on the left side of the chest was pricking in nature, non-radiating, and associated with palpitations. The cough was productive of clear sputum.

In the review of systems, she had no fever, diarrhea, nausea or vomiting. There was no history of weight changes reported or yellowness of the eyes. There had been no facial puffiness or changes in urine volume, color or frequency of micturition. She only had minimal straw-colored, non-foul-smelling per vaginal discharge. She didn’t report headache, confusion or loss of consciousness.

She previously had uneventful pregnancies except in 2005, her third pregnancy when she underwent an emergency caesarean section occasioned by antepartum hemorrhage due to placenta previa at 28 weeks of gestation, the outcome being a stillbirth. She then developed anemia for which she got blood transfusion and fully recovered. Her ante-natal care profile during the last pregnancy revealed that she was negative for HIV, syphilis, hepatitis B and that her hemoglobin was 11.2 g/dl and blood group O rhesus positive (O+). Her menarche was at 13 years and she had never used contraceptives her whole life.

Her previous hospital admissions were due to deliveries. She has no history of chronic illnesses (diabetes, hypertension, asthma etc) including any mental illness. She was therefore not on any medication. She had no known food or drug allergies. She was married and was a small scale farmer who had 6 living children.

### Clinical findings

She was in obvious respiratory distress as she was propped up and was using accessory muscles to breathe.. She had a respiratory rate of 35 cycles/minute and blood pressure of 133/65 mmHg. Her pulse was irregularly irregular at 86 beats/minute (bpm). Compared to her heart rate which was 126 bpm, she had a pulse deficit. She had no pallor, jaundice or lymphadenopathy. She had pitting edema of grade 4 (up to the sacral level). Her oxygen saturation was 89% on 5lts/min of oxygen.

She was conscious and well oriented. Her neck was soft, Kernig’s sign was negative and there were no cranial nerve palsies. She had an elevated jugular venous pressure. The trachea was central while the apex beat was displaced to the 6th intercostal space mid-clavicular line. The heart beats were irregularly irregular but there were no murmurs, rubs, clicks or other abnormal heart sounds. The chest was moving equally with respiration, had no areas of tenderness or masses, was resonant on percussion and with crepitations over the whole lung fields bilaterally. The abdomen was slightly distended in the suprapubic area but was moving with respiration. Bowel sounds were present. There was a tender hepatomegaly 8 cm below the costal margin mid-clavicular line. There was also hepatojugular reflux. The uterus was palpable at the level of the umbilicus, but not tender. The pelvic exam revealed normal genitalia, minimal non-foul smelling lochia, and a soft, central, non-tender cervix. She had normal anal opening, normal anal sphincter tone with no tenderness or masses, and fecal matter noted on examining finger during digital rectal exam.

She was thus diagnosed with congestive cardiac failure and atrial fibrillation due to peripartum cardiomyopathy, with differentials of pulmonary edema. There was a waxing and waning in her psychiatric symptoms and she was referred to the regional referral hospital where she succumbed a day after arrival. See the attached timeline of care.

## Discussion and conclusions

The above patient developed puerperal psychosis during the management of peripartum cardiomyopathy. It is rare for these two conditions to occur together. In addition, their co-occurrence complicates the management of the cardiac symptoms due to the adverse effects of antipsychotic medications on QTc which can cause sudden cardiac death (SCD) [13, 14].

Patients with PPCM face a high risk of life-threatening arrhythmias [15]. It has been estimated that arrhythmias contribute to nearly 19% of PPCM cases and markedly elevate the risk for SCD, particularly ventricular fibrillations and ventricular tachycardia [16]. In fact, a quarter of all deaths among women on management for PPCM are attributed to ventricular tachyarrhythmias [6]. A wearable cardioverter/defibrillator (WCD) has been demonstrated to prevent sudden arrhythmic death among PPCM patients [15].

Antipsychotic medications are a major adjunct in the management of puerperal psychosis [10, 12]. In a patient like the one presented herein, one must be aware of the clinical issues that are related to the cardiac management of a patient taking antipsychotic medications [13]. These issues range from sudden death, electrocardiogram (ECG) and corrected QT segment (QTc) changes in the short term (acute negative effects) and metabolic syndrome, diabetes, hyperlipidemia, and myocarditis in the long term [13]. QT, the time it takes for ventricular depolarization and repolarization, varies with heart rate changes. Thus QTc is often used and is calculated by dividing the measured QT segment by the square root of the R to R interval [17]. Normal QTc values are ≤0.44 s in men and ≤ 0.46 s in women, whereas QTc of > 0.50 s or an increase of 0.06 s from baseline is considered prolonged [18]. This effect is dose-dependent and is achieved by the antipsychotic blocking the repolarizing potassium currents [19]. This prolongation of the QTc is associated with increased risk for the life-threatening arrhythmia torsades de pointes (polymorphic ventricular tachycardia) [13].

Prolongation of the QTc is associated with hypokalemia, hepatitis C virus, HIV infection, T-wave abnormalities, female sex, age (70 years or older), agitation, and use of multiple QT prolonging medications [13]. The reported patient was female, agitated and on multiple QT prolonging medications (digoxin 0.25 mg once a day, furosemide 40 mg IV od, fluphenazine decanoate 25 mg IM stat, olanzapine 10 mg once a day). ECG monitoring is recommended for patients with cardiac risk factors; arrhythmia; if patient is on a second agent causing prolonged QTc; all patients starting the first-generation antipsychotic pimozide; and all patients admitted to an inpatient unit [20, 21]. Our patient needed regular QTc and electrolyte monitoring which was part of the reason for her referral. In case of a prolonged QTc, one must either decrease the dosage or discontinuation the antipsychotic medication (and institute alternative treatment with benzodiazepines, mood stabilizers, or electroconvulsive therapy (ECT) as alternatives to antipsychotic medications) [13].

In conclusion puerperal psychosis, a psychiatric emergency rarely occurs in the background of peripartum cardiomyopathy, itself a rare condition. If this happens as in the reported case the clinician must be cognizant and aware of the pitfalls of treatment of both conditions using antipsychotic medications. Alternative modes of therapy like ECT, benzodiazepines and mood stabilizers may be preferred. If however antipsychotics are to be used, there must be QTc monitoring using an ECG as it is associated with sudden cardiac death. Nevertheless, SCD may occur in patients on antipsychotics independent of any direct arrhythmogenic effects of the medications [22]. It would also be important to keep monitoring electrolytes.

## Supplementary information

**Additional file 1.** Timeline of Care

## Data Availability

Data sharing is not applicable to this article as no datasets were generated or analyzed during the current study.

## Abbreviations

^0^C: Degrees Celsius
bd: Twice daily
BP: Blood pressure
Bpm: Beats per minute
cm: Centimeters
DDx: Differential diagnosis
ECG: Electrocardiogram
ECT: Electroconvulsive therapy
Hb: Hemoglobin
HIV: Human immunodeficiency virus
IM: Intramuscular
iu: International unit
IV: Intravenous
MCH: Micro corpuscular hemoglobin
MCV: Micro corpuscular volume
mg: Milligrams
mmHg: Millimeters of mercury
mmol/l: Milimoles per litre
NBU: New born unit
od: Once daily
POCUS: Point of care ultrasound
PP: Postpartum, or puerperal, psychosis
PPCM: Peripartum cardiomyopathy
QTc: Corrected QT interval
QTc: Corrected QT segment
RBS: Random blood sugar
SCD: Sudden cardiac death
stat: At once or with no delay
WBCs: White blood cells
WCD: Wearable cardioverter/defibrillator
