# Supplementary material for: Postpartum psychosis in peripartum cardiomyopathy: a case report
Source: BMC Psychiatry. 2020 Mar 11;20:114. doi: 10.1186/s12888-020-02522-2 (PMC7066778; doi:10.1186/s12888-020-02522-2)
Supplement: Supplementary file 1 — Additional file 1. Timeline of Care [file 12888_2020_2522_MOESM1_ESM.docx]

**Additional Files**

**1. Timeline of Care**

This describes important information from the patient’s history, relevant physical examination and other significant clinical findings organized as a timeline.

| **Date** | **Relevant patient data** |
| --- | --- |
| Day 1 (admission) | Dyspnea, easy fatigability, orthopnea, paroxysmal nocturnal dyspnea, edema, tachycardia, tachypnea, irregularly irregular heart rate with a pulse deficit, cardiomegaly (displaced apex beat), hepatomegaly, pulmonary crepitations  Congestive cardiac failure with atrial fibrillation secondary to PPCM  Differential diagnosis: pulmonary edema;  Chest x-ray: enlarged cardiac shadow with pulmonary edema;  Full hemogram: WBCs: 14.9;lymphocytes: 4.31; granulocytes: 9.85; Hb: 12.2 g/dl; MCV and MCH were normal;  RBS: 7.5mmol/l;  Urinalysis: protein: nil; glucose: nil; nitrites: nil; leucocytes: nil; no deposits on microscopy;  ECG: irregularly irregular ventricular rate with fibrillatory waves; normal cardiac axis with no ST segment elevation;  Cardiac point of care ultrasound (POCUS): no pericardial effusion; irregular rhythm easily recognizable; all cardiac chambers were dilated but no intra chamber thrombus; reduction in ejection fraction to ~35%; pulmonary edema; liver was noted to be homogenously enlarged; there was no ascites visualized and the kidneys were of normal morphology and size;  Kidney function tests were requested while the patient was catheterized and iv lasix 40 mg od , digoxin 0.5mg stat then 0.25mg after 6 hours (until 1gm in 24 hours); iv heparin 6000iu bd and oral warfarin 5mg od was initiated;  Pediatric team review was requested for the baby because the woman was unable to breast feed; the baby had a fever of 39.5^0^C with a septic cord; the baby was taken to newborn unit (NBU) as the mother was allowed to stabilize; the mother was however encouraged to express milk; antibiotics were initiated and supplementary formula feeds provided as needed; |
| Day 2-4 | Marked improvement was reported as the dyspnea had reduced tremendously; patient became ambulant; there was no derangements noted in urinary output;  However, bilateral pitting edema of grade 3 was still noted;  BP was 103/77mmHg; pulse rate was 86 bpm and still irregularly irregular;  Chest: bilateral vesicular air entry;  Plan: continue with medication but stop heparin; |
| Day 5 | There was marked improvement in respiratory symptoms;  However, the woman refused to express milk and requested to have her baby;  The BP was 133/85mmHg, pulse 86, and there was vesicular breathing on auscultation;  She was transfered to post natal ward to be near baby and carvedilol 3.125mg bd was added to her treatment as she still had irregularly irregular pulse rate and a pulse deficit; |
| Day 6 at 1713hrs | Patient had shown signs of improvement but developed bizarre behavior and confusion; she also developed hallucinations (auditory, visual, olfactory-she claimed the baby had an offensive smell), violence to the baby and the husband, and refusal to feed and take medication; she also had tremors; there was no altered sensorium;  Body temperature was 36.5^0^C; respiratory rate was 17 cycles/minute; BP was 112/86mmHg; and pulse was 79 bpm and still irregular;  Other systems were unremarkable;  The full hemogram was normal and she was negative for malaria;  IM chlorpromazine 100mg stat then olanzapine 5mg od and trihexyphenidyl 5mg od was added to her medication to manage puerperal psychosis; |
| Day 7-11 | During this time she attempted to forcefully remove the urinary catheter, nasogastric tube and the peripheral IV line (branular); she still had hallucinations; was confused, violent, and with bizarre behavior;  The cardiac and respiratory symptoms had improved;  She continued with her medication; |
| Day 12 | Patient on management for peripartum cardiomyopathy and puerperal psychosis;  Cardiac complaints had resolved;  The patient still had hallucinations, confusion, violence and tremors.  Other system were unremarkable;  The plan was to: increase olanzapine to 10 mg od; administer IM fluphenazine decanoate 25 mgs stat; add carbamazepine 400mg stat then 200mg bd; and to continue with anti-failure drugs; |
| Day 13-14 | Patient reported improvement of both the cardiac and psychotic symptoms; with reduced episodes of hallucinations and violence; but she is still tremulous;  BP: 137/87mmHg; pulse 58bpm and still irregular;  At this point digoxin and warfarin were withheld; and the patient continued with furosemide, carvedilol, olanzapine, carbamazepine and trihexyphenidyl; |
| Day 15 | A decline was noted in the patient’s progress. The psychotic features started worsening even though the cardiac symptoms, apart from the irregularly heart rate, were controlled. Her visual, auditory and olfactory hallucinations were worsening. The bizarre behavior was back and she had attempted demonstrated violence to the baby again. There were no fevers or abnormalities in her vital signs. Nothing was significant on physical exam. She was therefore referred to the national referral hospital for further specialist management. However, on follow up, we were informed that she died a day after admission. A post-mortem was not done as the relatives declined. No obvious cause of death could be identified. |

NB:

1. Concerning the facilities where the patient was attended to: the emergency unit and the postnatal ward are within a Level IV hospital, also known as County Referral Hospital. At the National Referral Hospital, she was admitted to the Maternity Ward as she was still in puerperium.
2. The psychiatric consultation-liaison team first saw the patient on the 6th day of admission at the County Referral Hospital when she developed the psychiatric symptoms. They kept seeing her until she was referred to the National Referral Hospital.
